# Mathematics and Measurement

**DOI:** 10.6028/jres.106.011

**Published:** 2001-02-01

**Authors:** Ronald F. Boisvert, Michael J. Donahue, Daniel W. Lozier, Robert McMichael, Bert W. Rust

**Affiliations:** National Institute of Standards and Technology, Gaithersburg, MD 20899-0001

**Keywords:** deconvolution, digital libraries, history of NBS, linear algebra, mathematical reference data, mathematical software, micromagnetic modeling, parameter estimation, software testing, scientific computing

## Abstract

In this paper we describe the role that mathematics plays in measurement science at NIST. We first survey the history behind NIST’s current work in this area, starting with the NBS Math Tables project of the 1930s. We then provide examples of more recent efforts in the application of mathematics to measurement science, including the solution of ill-posed inverse problems, characterization of the accuracy of software for micromagnetic modeling, and in the development and dissemination of mathematical reference data. Finally, we comment on emerging issues in measurement science to which mathematicians will devote their energies in coming years.

## 1. Introduction

Mathematics plays an important role in the science of metrology. Mathematical models are needed to understand how to design effective measurement systems, and to analyze the results they produce. Mathematical techniques are used to develop and analyze idealized models of physical phenomena to be measured, and mathematical algorithms are necessary to produce practical solutions on modern computing devices. Finally, mathematical and statistical techniques are needed to transform the resulting data into useful information.

Applied mathematics has played a visible role at NBS/NIST since the Math Tables project in the 1930s, and formal mathematical and statistical organizations have been part of NBS/NIST since the establishment of the National Applied Mathematics Laboratory in 1947. Among these organizations was the NBS Institute for Numerical Analysis (1947–54), which has been credited as the birthplace of modern numerical analysis. The NIST Mathematical and Computational Sciences Division (MCSD) is the modern successor to these NBS/NIST organizations.

In this paper we indicate some of the important contributions of mathematics to NBS/NIST measurement programs during the past 60 years. We then provide examples of more recent efforts in the application of mathematics to measurement science. This includes work in the the solution of ill-posed inverse problems, characterization of the accuracy of software for micro-magnetic modeling, and in the development and dissemination of mathematical reference data. Finally, we comment on emerging issues in measurement science to which mathematicians will devote their energies in coming years.

## 2. History

### 2.1 Early Developments

Mathematical research at NBS began in the late 1930s when NBS Director Dr. Lyman J. Briggs conceived a project for the computation of tables of mathematical functions of importance in applications. The resulting Mathematical Tables Project was located in New York and administered by the Works Projects Administration. The project, under the direction of Arnold N. Lowan, employed mathematicians and a large number of additional staff to carry out the necessary computations by hand. From 1938 to 1946, 37 volumes of the NBS Math Tables Series were issued, containing tables of trigonometric functions, the exponential function, natural logarithms, probability functions, and related interpolation formulae [23].

Such tabulated values of mathematical functions can be considered to be the results of property measurements, though of a logical system rather than a physical one. Thus, the Bureau’s first foray into mathematical research was intimately involved with measurement.

The contributions of applied mathematics to the war effort in the 1940s fueled a widespread recognition of the importance of mathematical research to the attainment of national goals. In 1946, the Chief of Naval Research suggested that NBS consider the establishment of a national laboratory for applied mathematics. NBS Director Dr. Edward U. Condon was enthusiastic about the idea, and the National Applied Mathematics Laboratory (NAML) was established at NBS the following year with John H. Curtiss as its director. The program for the NAML was to have two main components: numerical analysis and statistical analysis [10]. The NAML had four main operating branches, the Institute for Numerical Analysis (INA), the Computation Laboratory, the Machine Development Laboratory, and the Statistical Engineering Laboratory. The first of these was housed at the University of California Los Angeles, while the remaining were located at NBS in Washington. These were the organizational beginnings of today’s Information Technology Laboratory at NIST, which continues to work in applied mathematics, statistics, and high performance scientific computation, among other areas.

The original prospectus for the NAML proposed that it serve as a computation center for the Federal government. Computing equipment for large-scale computations were not readily available in the late 1940s, of course. NAML was the first organization in the world to build and put into service a successful large scale, electronic, fully automatic stored-program digital computing system [10]. This system, the Standards Eastern Automatic Computer (SEAC), designed and built in collaboration with the NBS Electronics Division, was put into continuous operation in May 1950. Its original configuration included a 512-word mercury delay line memory and teletype input-output. Despite its staggering 12 000 diodes and 1000 vacuum tubes, the SEAC operated reliably 77 % of the time during its first three years of operation. A machine of somewhat different design, the Standards Western Automatic Digital Computer (SWAC), was built at the INA in Los Angeles. These unique computational facilities allowed mathematicians from NBS and other institutions to perform calculations that spurred the development of modern numerical analysis. The name NAML was dropped in 1954 in favor of “Applied Mathematics Division.”

### 2.2 Institute for Numerical Analysis

Approximately three-fourth’s of the output of NAML during its first 5 years was in numerical analysis. Research in this area was emphasized due to the surging need for appropriate mathematical methods for use in exploiting the nation’s emerging digital computing capability. Dr. Mina Rees, Director of the Mathematical Sciences Section of the Office of Naval Research, which provided more than 80 % of the funding for the NAML, is credited with this vision. The center of this activity within NAML was the INA, an organization that, in a very real sense, pioneered modern numerical analysis.

The list of INA Directors and permanent staff during its period of operation (1947–54) reads like a Who’s Who of modern numerical analysis, including Forman S. Acton, George E. Forsythe, Magnus R. Hestenes, Fritz John, Cornelius Lanczos, Derrick H. Lehmer, J. Barkeley Rosser, Charles B. Tompkins, and Wolfgang R. Wasow. These were augmented by many visiting faculty appointments, junior researchers, and graduate fellows.

Among INA’s areas of emphasis were:

- Solution of linear systems of equations
- Linear programming
- Computation of eigenvalues of matrices
- Finite difference methods for partial differential equations
- Monte Carlo methods
- Numerical solution of ordinary differential equations
- Numerical methods for conformal mapping
- Asymptotic expansions
- Interpolation and quadrature

The story of the development of Krylov subspace methods for the solution of systems of linear algebraic equations illustrates the far-reaching impact of the INA’s technical program. The conjugate gradient algorithm is the earliest example of this class of methods. It is a method of iterative type which does not require explicit storage or manipulation of a matrix (only the ability to apply the underlying operator to any given vector). As such, it is ideal for the solution of very large and sparse systems. For symmetric (or Hermitian) positive definite systems it has the property that it converges in finite time (after *n* iterations for a system of order *n*); nevertheless, because of its iterative nature it can often be stopped earlier, providing acceptable results at moderate cost.

The first complete description of the conjugate gradient method appeared in a paper published in the NBS Journal of Research by Magnus R. Hestenes and Eduard Stiefel [19]. Hestenes was a member of the NBS INA, and Stiefel a visiting researcher from the Eidgenössischen Technischen Hochschule (ETH) in Zurich. Their paper remains the classic reference on this method. Other INA staff, such as Cornelius Lanczos and Marvin Stein, also made fundamental early contributions to the development of the method.

While there was much early interest in the algorithm, it went into eclipse in the 1960s as naive implementations failed on the increasingly larger problems which were being posed. Interest in the conjugate gradient method surged again in the 1970s when researchers discovered new variants and successful techniques for preconditioning the problem (i.e., premultiplication by a carefully chosen easily invertible matrix) to reduce the number of iterations. Today, these methods are the standard techniques employed for the solution of large linear systems. Citation searches for the term conjugate gradient turn up more than one million articles in which the term is used during the last 25 years. Krylov subspace methods were identified as one of the top ten algorithms of the century by *Computing in Science and Engineering*^1^ [13] in January 2000. An account of the history of the conjugate gradient method can be found in Ref. [18].

NBS was required to give up the administration of the INA in June 1954, a result of new Department of Defense rules which torpedoed funding arrangements with the Office of Naval Research. This was one of the unfortunate events in the wake of the AD-X2 battery additive controversy in which NBS found itself embroiled from 1948 to 1953. The INA was transferred to UCLA, but by this time most INA members had taken positions in industry and in universities. More information about the INA can be found in the accounts of Todd and Hestenes [20, 34].

### 2.3 Handbook of Mathematical Functions

With the establishment of the NAML in 1947 the Math Tables Project was transferred to the NBS Computation Laboratory. Subsequent tabulations were issued in the newly established NBS Applied Mathematics Series (AMS) of monographs, whose earliest issue provided tables of Bessel functions [2].

In 1956 NBS embarked on another ambitious program which was a natural outgrowth of its work on mathematical tables. Led by Dr. Milton Abramowitz, who was then Chief of the Computation Laboratory, the project would develop a compendium of formulas, graphs, and tables which would provide practitioners with the most important facts needed to use the growing collection of important mathematical functions in applications. Among these are the Bessel functions, hypergeometric functions, elliptic integrals, probability distributions, and orthogonal polynomials.

With substantial funding from the National Science Foundation, many well-known experts in the field were enlisted as authors and editorial advisors to compile the technical material. By the summer of 1958, substantial work had been completed on the project. Twelve chapters had been written, and the remaining ones were well underway. The project experienced a shocking setback one weekend in July 1958 when Abramowitz suffered a fatal heart attack. Irene Stegun, the Assistant Chief of the Computation Laboratory, took over management of the project. The exacting work of assembling the many chapters, checking tables and formulas, and preparing material for printing took much longer than anticipated. Nevertheless, the 1046-page *Handbook of Mathematical Functions, with Formulas, Graphs, and Mathematical Tables* was finally issued as AMS Number 55 in June 1964 [1].

The public reaction to the publication of the Handbook was overwhelmingly positive. In a preface to the ninth printing in November 1970, NBS Director Lewis Branscomb wrote

The enthusiastic reception accorded the “Handbook of Mathematical Functions” is little short of unprecedented in the long history of mathematical tables that began when John Napier published his tables of logarithms in 1614.

The Handbook has had enormous impact on science and engineering. The most widely distributed NBS/NIST technical publication of all time, the government edition has never gone out of print (more than 145 000 have been sold), and it has been continuously available as a Dover reprint since 1965. The Handbook’s citation record is also remarkable. More than 23 000 citations have been logged by Science Citation Index (SCI) since 1973. Remarkably, the number of citations to the Handbook continues to grow, not only in absolute numbers, but also as a fraction of the total number of citations made in the sciences and engineering. During the mid 1990s, for example, about once every 1.5 hours of each working day some author, somewhere, made sufficient use of the Handbook to list it as a reference.

### 2.4 Mathematical Analysis

A number of difficult mathematical problems emerged in the course of developing the Handbook which engaged researchers in the Applied Mathematics Division for a number of years after its publication. Two of these are especially noteworthy, the first having to do with stability of computations and the second with precision.

Mathematical functions often satisfy recurrence relations (difference equations) that can be exploited in computations. If used improperly, however, recurrence relations can lead to ruinous errors. This phenomenon, known as instability, has tripped up many a computation that appeared superficially to be straightforward. The errors are the result of subtle interactions in the set of all possible solutions of the difference equation. Frank W. J. Olver, who wrote the Handbook’s chapter on Bessel functions of integer order, studied this problem extensively. In a paper published in 1967 [28], Olver provided the first (and only) stable algorithm for computing all types of solutions of a difference equation with three different kinds of behavior: strongly growing, strongly decaying, and showing moderate growth or decay. This work is reflected today in the existence of robust software for higher mathematical functions.

Another important problem in mathematical computation is the catastrophic loss of significance caused by the fixed length requirement for numbers stored in computer memory. Morris Newman, who co-authored the Handbook’s chapter on combinatorial analysis, sought to remedy this situation. He proposed storing numbers in a computer as integers and performing operations on them exactly. This contrasts with the standard approach in which rounding errors accumulate with each arithmetic operation. Newman’s approach had its roots in classical number theory: First perform the computations modulo a selected set of small prime numbers, where the number of primes required is determined by the problem. These computations furnish a number of local solutions, done using computer numbers represented in the normal way. At the end, only one multilength computation is required to construct the global solution (the exact answer) by means of the Chinese Remainder Theorem. This technique was first described in a paper by Newman in 1967 [26]; it was employed with great success in computing and checking the tables in Chap. 24 of the Handbook. Today, this technique remains a standard method by which exact computations are performed.

In the 1960s NBS mathematicians also made pioneering efforts in the development and analysis of graph-theoretic algorithms for the solution of combinatorial optimization problems. Jack Edmonds did ground-breaking work in the analysis of algorithms and computational complexity, focusing on the establishment of measures of performance which distinguished practical algorithms from impractical ones [15]. This work provided a solid foundation for algorithms which have become the mainstay of operations research. In recognition of this work, Edmonds received the 1985 John Von Neumann prize from the Institute for Operations Research and the Management Sciences (INFORMS).

### 2.5 Scientific Computing

As computing systems became more powerful, NBS scientists were increasingly drawn to the use of computational methods in their work. Results of experimental measurements needed to be analyzed, of course, but more and more scientists were using mathematical models to help guide the measurement process itself. Models could be developed to simulate experimental systems in order to determine how best to make the measurements or how to correct for known systematic errors. Finally, mathematical models could be used to understand physical systems that were extremely difficult to measure. Increasingly, NBS mathematicians were being consulted to help develop such models and to aid in devising computational methods of solving them.

While the NBS Applied Mathematics Division had always engaged in collaborative work with NBS scientists, during the 1970s through the 1990s this became the central theme of its work (and that of its successor organizations, the Center for Applied Mathematics, and then the Computing and Applied Mathematics Laboratory). Examples of such long-term collaborations include the study of combustion, smoke and gas flow during fires [4], the modeling of semiconductor devices [5], and the modeling of alloy solidification processes [9].

In the 1980s the newly formed NBS Scientific Computing Division began the development of a repository of mathematical software tools to aid NBS scientists in the development and the solution of models. Among these were the NIST Core Mathematics Library (CM-LIB) and the joint DOE/NIST Common Math Library (SLATEC) [8]. The growing collection of such tools was indexed in the Guide to Available Mathematical Software (GAMS) [6], which continues to provide the computational science community with information on, and access to, a wide variety of tools, now via a convenient Web interface (http://gams.nist.gov/).

### 2.6 Current Mathematical Research

Today the NIST Mathematical and Computational Sciences Division (MCSD) is focused on (1) assuring that the best mathematical methods are applied in solving technical problems of the NIST Laboratories, and (2) targeted efforts to improve the environment for computational science within the broader research community. The Division provides expertise in a wide variety of areas, such as nonlinear dynamics, stochastic methods, optimization, partial differential equations (PDE), computational geometry, inverse problems, linear algebra, and numerical analysis. This is applied in collaborative research projects performed in conjunction with other NIST Laboratories. Substantial modeling efforts are underway in the analysis of the properties of materials, in computational electromagnetics, and in the modeling of high-speed machine tools, for example. Modeling efforts are supported by work in the development of mathematical algorithms and software in areas such as adaptive solution of PDEs, special functions, Monte Carlo methods, deconvolution, and numerical linear algebra.

In response to needs of the wider community, MCSD has developed a number of Web-based information services, such as the Guide to Available Mathematical Software, the Matrix Market (see Sec. 4.1), the Java Numerics site, and the Digital Library of Mathematical Functions (see Sec. 5). In addition, staff members are involved in standardization efforts for fundamental linear algebra software and for numerical computing in Java.

Current work of the Division is described in its Web pages at http://math.nist.gov/. The following sections provide further details of several of these projects which have particular relevance to the measurement sciences.

## 3. Mathematics of Physical Metrology

In physical metrology it is often necessary to fit a mathematical model to experimental results in order to recover the quantities being measured. In some cases the desired variables can be measured more or less directly, but the measuring instruments distort the measured function so much that mathematical modeling is required to recover it. In other cases the desired quantities cannot be measured directly and must be inferred by fitting a model to the measurements of variables dynamically related to the ones of interest.

### 3.1 Deconvolution

An example of measurements of the first type was brought to Bert Rust’s attention by Jeremiah Lowney [24] of the NIST Semiconductor Electronics Division. The measurements were linear scans by a scanning electron microscope (SEM) across a semiconductor chip on which a circuit had been etched. The goal was to measure the location of features on the chip with uncertainty levels of 10 nanometers (nm) or smaller. The measurements are modeled by a system of linear, first kind integral equations,

$$\begin{array}{c} y_{0}(x_{i})=\int_{x_{i}-4\sigma }^{x_{i}+4\sigma }K(\xi -x_{i})y_{t}(\xi )d\xi +\epsilon_{i},\\ i=1,2,\ldots ,301, \end{array}$$ (1)

where the variables *x* and *ξ* are both distances (in nm) along the scan, *y*_t_(*ξ*) is the desired “true” signal strength at distance *ξ*, and the values *y*_0_(*x_i_*) are observed signal strengths on a mesh *x*_1_, *x*_2_, …, *x*_301_, with a mesh-width **Δ***x* = *x_i_*_+1_ − *x_i_* = 2 nm. These measured values fail to give a faithful discrete representation of the unknown function *y*_t_(*ξ*) because they are smoothed by the measurement process and because of the additive random measurement errors **ϵ***_i_*. The incident scanning beam is not infinitely sharp. The beam intensity is thought to have a Gaussian profile

$$K(\xi -x_{i})=\frac{1}{\sqrt{2\pi }\sigma }\exp\left\{-\frac{1}{2\sigma^{2}}{\left(\xi -x_{i}\right)}^{2}\right\},$$ (2)

with a “beam diameter” *d* = 2.56***σ*** = 37.5 nm. The observed signal is thus the sum of a convolution of the true signal *y*_t_(*ξ*) with this Gaussian function and the random measuring errors.

**Fig. 1:**
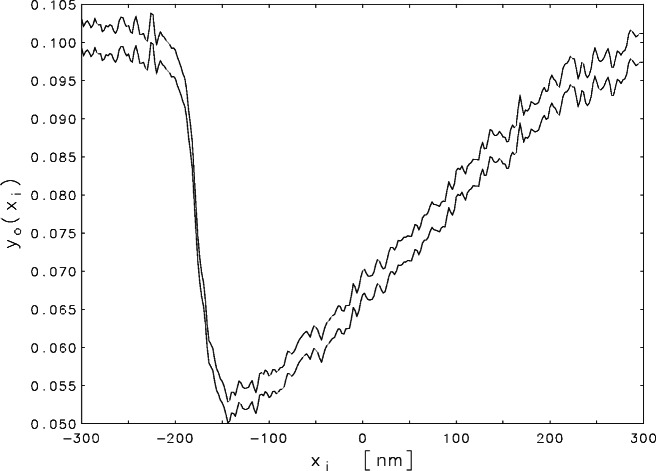
Upper and lower 95 % confidence bounds for the observed signal from the SEM plotted as a function of distance *x* (in nanometers) on the chip, with the zero chosen to be at the center of the record.

The measurements for a scan across a sharp step-like edge are shown in Fig. 1. At the left of the plot, the electron beam is incident on (and perpendicular to) a presumably flat surface. The electrons penetrate the semiconductor and excite a roughly spherical distribution of secondary emissions. Most of these secondary electrons are reabsorbed by the material but a significant fraction escape into the vacuum chamber above. These escaped electrons are collected by an electrode to generate the current that gives the measured signal. As the primary beam crosses the edge, more and more of the emitted electrons come from the lower surface, and many of these are reabsorbed by the wall. Thus there is a sharp drop in the signal. Even when the incident beam has moved well clear of the wall, its “shadow” persists for a large distance, causing a slow recovery of the signal to its original level.

To estimate *y*_t_(*ξ*) it is necessary to discretize the integral equations to obtain an underdetermined linear regression model

$$y_{o}=Ky_{t}+\epsilon ,\;\epsilon \sim N(0,S^{2}),$$ (3)

where ***y***_o_ and **ϵ** are order-301 vectors containing the known measurements and unknown measuring errors, ***K*** is a known matrix with elements *K_i,j_* = *K_i_*(*ξ_j_*)**Δ***ξ*, ***S***^2^ is the (estimable) variance matrix for the errors, and ***y***_t_ is an unknown vector of length 361 whose elements comprise a discrete approximation to *y*_t_(*ξ*) on a mesh *ξ*_1_, *ξ*_2_, …, *ξ*_361_ with mesh spacing **Δ***ξ* = **Δ***x*. The limits of each integral extend for a distance of 4***σ*** = 1.5625*d* nm on each side of the corresponding measurement point *x_i_*. For the middle 301 points in the discretization mesh *ξ_i_*_+30_ = *xi*, but 30 extra *ξ_j_* points were required on each end of the range [*x*_1_, *x*_301_] to accommodate these limits of integration. This means that the linear regression model has more unknowns than equations, with the dimensions of ***K*** being 301 × 361. This indeterminacy, which is common in deconvolution problems, admits an infinitude of least squares estimates which solve the problem equally well, but almost all of them are physically impossible.

**Fig. 2:**
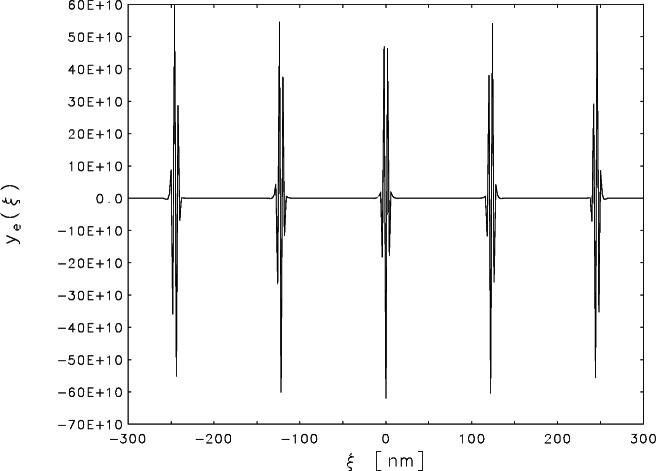
Linear least squares estimate of ***y***_t_, assuming fixed constant extensions outside the range of measurements, plotted as a function of distance *ξ* (in nanometers) from the center of the record.

There are many ways to make the problem exactly determined (i.e., to transform ***K*** into a square matrix) by making assumptions about the behavior of *y*_t_(*ξ*) outside the range of measurements. But the resulting square matrix is always ill conditioned so the least squares estimate, though unique, always oscillates wildly between extreme positive and negative values. Figure 2 is a plot of the estimate *y*_e_(*ξ*) when it is assumed that

$$\begin{array}{cc} y_{t}(\xi_{j})=y_{t}(\xi_{31}), & j=1,2,\ldots ,30,\\ y_{t}(\xi_{j})=y_{t}(\xi_{331}), & j=332,333,\ldots ,361, \end{array}$$ (4)

which reduces the number of unknowns from 361 to 301. The flat looking segments of the curve are actually oscillating between extreme values on the order of ±10^6^. To understand this behavior it is necessary to consider the effect of the measurement errors on the estimate.

The measured data did not come with uncertainty estimates, but before the scan reached the edge, there was a long segment (not shown in Fig. 1) where the beam was moving over a flat surface, so all of the variations could be attributed to measurement errors. By analyzing those variations and using the theoretical knowledge that the standard deviation of the error should be proportional to the square root of the signal strength, it was possible to estimate a variance matrix ***S***^2^ for the observations. The errors at adjacent mesh points were correlated, so ***S***^2^ was not diagonal, but it was positive definite, so it had a Cholesky factorization ***S***^2^ = ***LL***^⊤^, where ***L*** is a lower triangular matrix. Scaling the regression model with ***L***^−1^ gives

$$\begin{array}{c} L^{-1}y_{o}=L^{-1}Ky_{t}+L^{-1}\epsilon ,\\ L^{-1}\epsilon \sim N(0,I_{301}), \end{array}$$ (5)

where ***I***_301_ is the order-301 identity matrix. The fact that the random errors in this rescaled model are independently distributed with the standard normal distribution simplifies the analysis of the effects of those errors on the estimated solution.

Let ***y***_e_ be an estimate for ***y***_t_ and

$$r=L^{-1}(y_{o}-Ky_{e})$$ (6)

be the corresponding residual vector. Comparing this expression with Eq. (5) suggests that ***y***_e_ is acceptable only if ***r*** is a plausible sample from the (***L***^−1^***ϵ***)-distribution. This means that the elements of ***r*** should be distributed *N*(0,1), and the sum of squared residuals ***r***^⊤^***r*** should lie in some interval $\left[301-\kappa \sqrt{602},\;301+\kappa \sqrt{602}\right]$, with |***κ***|< 2. This last condition follows from the fact that

$$\sum_{i=1}^{301}{\left(L^{-1}\epsilon \right)}_{i}^{2}=\epsilon^{⊤}S^{-2}\epsilon \sim \chi^{2}(301).$$ (7)

hence

$$\varepsilon \{\epsilon^{⊤}S^{-2}\epsilon \}=301,\;\text{Var}\{\epsilon^{⊤}S^{-2}\epsilon \}=2\times 301.$$ (8)

When the assumptions of Eq. (4) are imposed on the scaled model of Eq. (5)***L***^−1^***K*** becomes a 301 × 301 matrix, so, in theory, the unique least squares estimate satisfies ***L***^−1^***y***_o_ = ***L***^−1^***Ky***_e_ exactly. Because of rounding errors, calculations on a real computer did not give an exact **0** residual vector, so the calculated sum of squared residuals was 8.20 × 10^−4^ which is neglible when compared to the expected value 301. This means that almost all of the variance in the measured record is explained by the model. A significant part of that variance is due to measurement errors, so the least squares estimate has captured variance that properly belongs in the residuals. This misplaced variance is amplified by the ill-conditioning to produce the wild oscillations in Fig. 2.

One approach to resolving the indeterminacy in Eq. (5) and stabilizing the estimated solution is to impose physically motivated *a priori* constraints in order to reduce the size of the set of feasible solutions. For many measurement problems, nonnegativity is an appropriate and often powerful constraint, especially when computing confidence intervals for the estimate. Consider the case of computing upper and lower confidence bounds for each of the 361 elements of the estimated solution. Let the chosen confidence level be 100*α*% (with 0 < *α* < 1), and define

$${\left\|L^{-1}(y_{o}-\mathrm{Ky})\right\|}^{2}={\left(y_{o}-\mathrm{Ky}\right)}^{⊤}S^{-2}(y_{o}-\mathrm{Ky}).$$ (9)

The problem then is, for *j* = 1, 2, …, 361, to compute

$${\hat{y}}_{j}^{\text{lo}}=\underset{y\geq 0}{\min}\left\{e_{j}^{⊤}y|{\left\|L^{-1}(y_{0}-\mathrm{Ky})\right\|}^{2}=\mu^{2}\right\},$$ (10)

$${\hat{y}}_{j}^{\text{up}}=\underset{y\geq 0}{\max}\left\{e_{j}^{⊤}y|{\left\|L^{-1}(y_{0}-\mathrm{Ky})\right\|}^{2}=\mu^{2}\right\},$$ (11)

where ***e****_j_* is the unit vector whose *j*th element is one, and *μ*^2^ is a statistical parameter which must be chosen to guarantee that

$$\mathrm{Pr}\{{\hat{y}}_{j}^{\text{lo}}\leq e_{j}^{⊤}y_{t}\leq {\hat{y}}_{j}^{\text{up}}\}\geq \alpha .$$ (12)

In 1972, Rust and Burrus [32] conjectured, and in 1994 Rust and O’Leary [33] proved that valid 100*α* % confidence intervals are obtained if

$$\mu^{2}=\rho_{\min}+\kappa^{2},$$ (13)

where

$$\rho_{\min}=\underset{y\geq 0}{\min}\left\{{\left\|L^{-1}(y_{o}-\mathrm{Ky})\right\|}^{2}\right\},$$ (14)

and ***κ*** is the *α*-percentile for the *N*(0,1) distribution.

The calculation of each of the bounds in Eqs. (10) and (11) is a separate quadratic programming problem. In 1972, Rust and Burrus [32] gave heuristic arguments to show that each pair ${\hat{y}}_{j}^{\text{lo}}$ and ${\hat{y}}_{j}^{\text{up}}$ were the two roots of the piecewise quadratic equation

$${ℒ}_{j}(\psi )=\underset{y\geq 0}{\min}\left\{{\left\|L^{-1}(y_{o}-\mathrm{Ky})\right\|}^{2}|e_{j}^{T}y=\psi \right\}.$$ (15)

In 1986 O’Leary and Rust [27] gave a formal proof of this fact and presented an efficient algorithm called BRAKET-LS for calculating those roots. It has been successfully used for radiation spectrum unfolding by users both at NIST [14] and other laboratories [16].

**Fig. 3:**
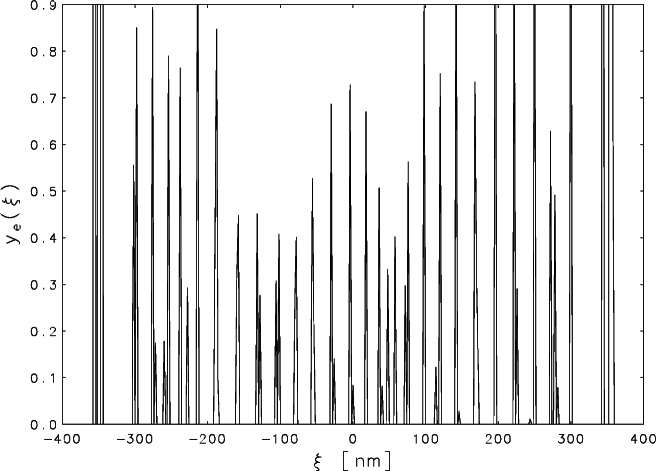
Constrained linear least squares estimate, with constraints *y_j_* α;0.045, *j* = 1, 2, …, 361, plotted as a function of distance α; (in nanometers) from the center of the record.

An inspection of Fig. 1 reveals that, for the present problem, the constraints *y_j_* ≥ 0.045 are even more appropriate than nonnegativity. These constraints can be reduced to nonnegativity by a simple transformation of variables, but unfortunately, as indicated by Fig. 3, they do not constrain the solution set enough to overcome the indeterminacy. The vertical axis has been truncated in order to exhibit the behavior of the estimate in the interval −300 ≤ *ξ* ≤ 300. The maximum *y*_e_(*ξ*) would have to be increased to the value 70.0 to accommodate the off-scale excursions on both ends of the plot.

**Fig. 4:**
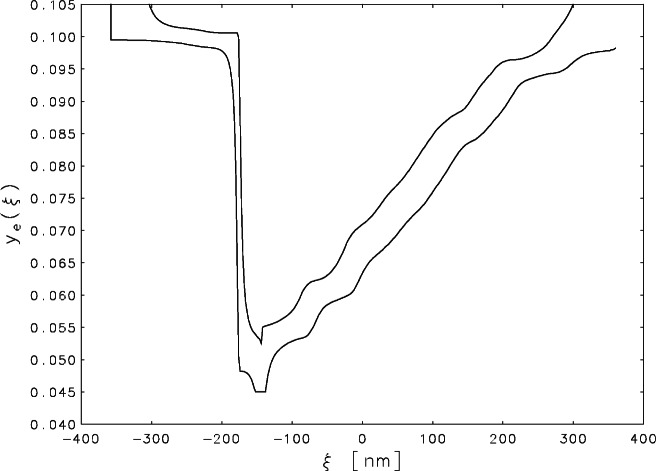
Monotonicity constrained, one-at-a-time, 95 % confidence interval bounds for the true signal plotted as a function of the distance *ξ* (in nanometers) from the center of the record.

Fortunately, there are more powerful constaints which are appropriate for this problem and which can be reduced to nonnegativity by a transformation of variables. For a simple edge, the signal should be monotonically non-increasing before it “bottoms out” and monotonically non-decreasing during the recovery. It is easy to design a matrix ***T*** so that the linear transformation ***y***_t_ = ***Tz*** converts the constraints ***z*** ≥ **0** into the desired monotonicity constraints on the elements of ***y***_t_. One can then use BRAKET-LS on the transformed problem with unknown solution ***z***. Figure 4 gives the upper and lower two standard deviation bounds obtained when the turning point is chosen to be *ξ*_109_ = −144 nm. The bounds explode at each end of the record, but within the interval of measurement [−300, +300] nm, the confidence intervals are comparable in size to those for the measured signal (cf. Fig. 1). The most difficult part of designing the matrix ***T*** is determining the point where the non-increasing constraint should be changed to a non-decreasing one. If a gap is left between the two segments, the uncertainties explode in that gap, but the bounds in the two segments are not very sensitive to the small variations in the turning point, so good results can be obtained by trial and error.

**Fig. 5:**
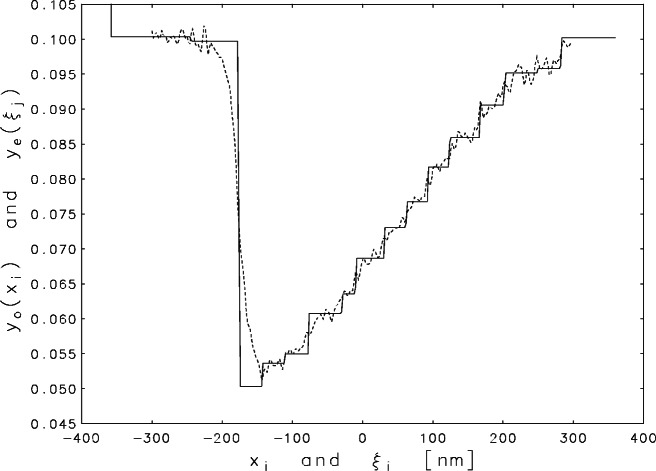
Monotonicity constrained estimate (solid curve) and observed signal (dashed curve) plotted as functions of distance (in nanometers) from the center of the record.

The quality of the confidence intervals in Fig. 4 is a good recommendation for the corresponding estimate which is plotted in Fig. 5. The estimate is plotted as a solid curve and the measured data as a dashed curve. The edge definition for the estimate is much sharper than that for the data. The sharp drop begins at *ξ*_92_ = −178 nm and reaches the minimum level at *ξ*_94_ = −174 nm. The drop is almost, but not exactly, linear. The single intermediate point at *ξ*_93_ = 176 nm falls slightly to the left of the straight line connecting the two extreme points. The uncertainties indicated in Fig. 4 are not sufficiently small to permit the conclusion that the variation in the interval −178 ≤ *ξ* ≤ −174 represents a real departure from a vertical drop rather than an uncertainty in the location of that drop. In the latter case, the results indicate that the best estimate for that location is (176 ± 2) nm, and in either case the original goal of measuring the location of the drop to an accuracy of 10 nm or smaller has been attained.

A surprise in the estimate in Fig. 5 was the staircase form of the recovery segment. This is unlikely to be the result of an inappropriate assumption of monotonicity. Had the monotonicity assumptions not been correct, the likely result would have been null sets for the feasible regions in the constrained estimation problems of Eqs. (10) and (11). The bounds in Fig. 4, which were calculated independently from the estimate, also display a hint of this staircase effect. One should not dismiss the idea that the steps indicate a real layering of the material in the etched chip. Studies with scanning tunneling microscopes [17, 21] of etched surfaces on silicon have revealed terraces with width distributions very similar to the distribution of widths of the steps in the figure. But more work should be done before drawing any conclusions about the cause of these steps because the interpretation of SEM scans is not simple or easy.

### 3.2 Parameter Estimation

Another important mathematical modeling problem in physical metrology is fitting a system of ordinary differential equations (ODEs) to a set of observed time series data which are corrupted by measurement errors. The desired quantities, which are unknown parameters in the ODEs, cannot be measured directly. Their values must be inferred from the fit to the measurements of dynamically related variables.

An example of a problem like this was brought to Bert Rust by Robert W. Ashton of the NIST Biotechnology Division. The problem arose in connection with a study of the ability of anhydrothrombin (AT), a derivative of the enzyme thrombin (T), to compete with thrombin for the binding of a potent thrombin inhibitor hirudin (H). The chemical equations for the reactions are

$$\begin{array}{ccccc} & & \underset{+}{\left(\text{AT}\right)} & & \\ T & + & H & \overset{\alpha_{2}}{\underset{\alpha_{1}}{⇄}} & \left(\text{TH}\right)\\ & & \alpha_{4}\underset{\left(\text{ATH}\right)}{↓↑}\alpha_{3} & & \end{array}$$

where (TH) is the thrombin-hirudin complex, (ATH) is the anhydrothrombin-hirudin complex, and *α*_1_, *α*_2_, *α*_3_, and *α*_4_ are the rates of the indicated reactions.

**Fig. 6:**
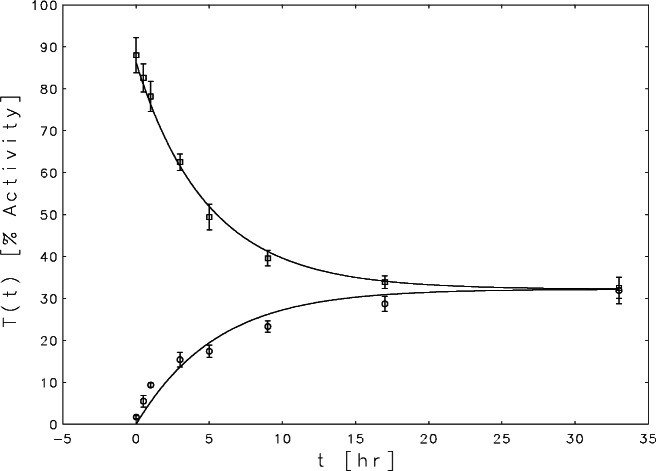
Simultaneous fits to the two thrombin concentration time series.

Two experiments were performed. In the first thrombin and hirudin were allowed to react, forming the (TH) complex, until equilibrium was established, and then, at time *t* = 0, an aliquot of anhydrothrombin was added. Then, over the next 33 hours, aliquots were removed at eight unequally spaced times and assayed for thrombin activity. This experiment was repeated three times, so the final data consisted of an average value and an estimate of its uncertainty at each of the eight time points. These data are are plotted on the upper curve in Fig. 6 with the concentrations of thrombin in units of percent activity.

In the second experiment, anhydrothrombin and hirudin were allowed to come to equilibrium with the (ATH) complex, and at time *t* = 0 an aliquot of thrombin was added. Then again, thrombin assays were taken at eight unequally spaced times. This experiment was also repeated three times to get averages and uncertainties. These data are plotted on the lower curve in Fig. 6. Note that the thrombin concentrations in the two experiments converge to the same equilibrium value.

The mathematical problem is to estimate *α*_1_, *α*_2_, *α*_3_, and *α*_4_ from the 16 measured thrombin concentrations. It can be shown that

$$\alpha_{4}=\beta \frac{\alpha_{2}\alpha_{3}}{\alpha_{1}},$$ (16)

where *β* = 0.4312, so the ODEs describing the kinetics of the reactions can be written

$$\begin{array}{c} \frac{dT}{dt}=\alpha_{1}(\text{TH})-\alpha_{2}(T)(H),\\ \frac{d(\text{AT})}{dt}=\alpha_{3}(\text{ATH})-\beta \frac{\alpha_{2}\alpha_{3}}{\alpha_{1}}(\text{AT})(H),\\ \frac{\text{dH}}{dt}=\alpha_{1}(\text{TH})+\alpha_{3}(\text{ATH})\\ -\alpha_{2}(T)(H)-\beta \frac{\alpha_{2}\alpha_{3}}{\alpha_{1}}(\text{AT})(H),\\ \frac{d(\text{TH})}{dt}=\alpha_{2}(T)(H)-\alpha_{1}(\text{TH}),\\ \frac{d(\text{ATH})}{dt}=\beta \frac{\alpha_{2}\alpha_{3}}{\alpha_{1}}(\text{AT})(H)-\alpha_{3}(\text{ATH}). \end{array}$$ (17)

Ashton and his colleagues [35] had already found an approximate solution to this problem using two perturbation expansions, but they wanted an independent confirmation of their result.

Equations (17) are nonlinear ODEs which do not have a closed form solution. To estimate the vector ***α*** = (*α*_1_, *α*_2_, *α*_3_)^⊤^ it is necessary to combine a numerical ODE integrator with a nonlinear fitting program to minimize the sum of squared residuals

$$ℒ(\alpha )=\sum_{k=1}^{2}\sum_{i=1}^{8}{\left[T_{k,i}^{c}(\alpha )-T_{k,i}^{o}\right]}^{2},$$ (18)

where *k* is the experiment number, *i* is the index of measurement times, the $T_{k,i}^{o}$ are the measured values, and the $T_{k,i}^{c}(\alpha )$ are the corresponding predicted values obtained by numerically integrating the ODEs.

The nonlinear fitting program begins with initial estimates ***α***^(0)^ and iterates to a local minimum of Eq. (18). On each iteration it must integrate the system [Eq. (17)] with the current values of ***α***. It also requires the partial derivatives $\frac{\partial ℒ}{\partial \alpha_{1}}$, $\frac{\partial ℒ}{\partial \alpha_{2}}$, and $\frac{\partial ℒ}{\partial \alpha_{3}}$ in order to compute the step for the next iteration. To obtain these, it must also integrate the system of 15 *variational equations* obtained by taking partial derivatives of each of the ODEs in Eq. (17) with respect to *α*_1_, *α*_2_, and *α*_3_.

Fortunately the initial values for the solutions to the ODEs in Eq. (17) were known exactly. For many problems of this type, the initial values are either unknown or are measured values, subject to the same kind of measurement errors as the other measured points. In such cases, it is necessary to treat them as unknown parameters to be determined by the fit. The fitting problem is then much more difficult. Even for relatively simple problems like the present one, the response function [Eq. (18)] has many local minima corresponding to values of ***α*** which do not give good fits to the data. It is absolutely necessary to pick starting estimates ***α***^(0)^ close enough to the correct local minimum to give a good fit. The difficulty of finding such values increases very rapidly as the number of unknown parameters increases.

The estimates obtained by simultaneously fitting the data from the two experiments were

$$\begin{array}{l} {\hat{\alpha }}_{1}=(1.62\pm .23)\times 10^{-5}s^{-1},\\ {\hat{\alpha }}_{2}=(6.0\pm 1.6)\times 10^{7}s^{-1},\\ {\hat{\alpha }}_{3}=(3.01\pm .31)\times 10^{-5}s^{-1}. \end{array}$$

The corresponding solutions to the ODEs are plotted as smooth curves in Fig. 6. The fits accounted for 99.4 % of the combined total variance in the two measured records. These results agree quite well with those obtained by Ashton and his colleagues, so the goal of the exercise was attained. But it should be noted that the lower curve does not fit its data as well as the upper curve, and that there is no guarantee that there is not another local minimum of Eq. (18) which would give even better fits.

## 4. Testing and Evaluation of Software

The need for measurement as an aid to understanding is not unique to physical systems. As software systems increase in complexity, many of their properties have become difficult to know a priori. Thus, experimental techniques for evaluating performance characteristics of software, such as speed and accuracy, have come into widespread use. We will describe several recent projects which are providing tools for the measurement of properties of mathematical algorithms and software.

A frequently applied method for the testing of numerical software is to exercise it on a battery of representative problems. Often such problems are generated randomly, insuring that a large number of test cases can be applied. Unfortunately, this is rarely sufficient for serious numerical software testing. Errors or numerical difficulties typically occur for highly structured problems or for those near to the boundaries of applicability of the underlying algorithm. These parts of the domain are rarely sampled in random problem generation, and hence testing must also be done on problem sets that illustrate particular behaviors. These are often quite difficult to produce, and, thus, researchers often exchange sample problem sets. Such data sets serve a variety of additional purposes:

1. Defining the state-of-the-art.
2. Characterizing industrial-grade applications.
3. Catalyzing research by posing challenges.
4. Providing a baseline of performance for software developers.
5. Providing data for users who want to gain confidence in software.

Unfortunately, these collections are often lost when the underlying technology is picked up by the commercial sector, leaving software developers and users without an important tool to use in judging the capability of their products. In this section we will describe recent work in the NIST Mathematical and Computational Sciences Division to address such needs in core linear algebra software and in micromagnetic modeling software.

### 4.1 The Matrix Market

**Fig. 7:**
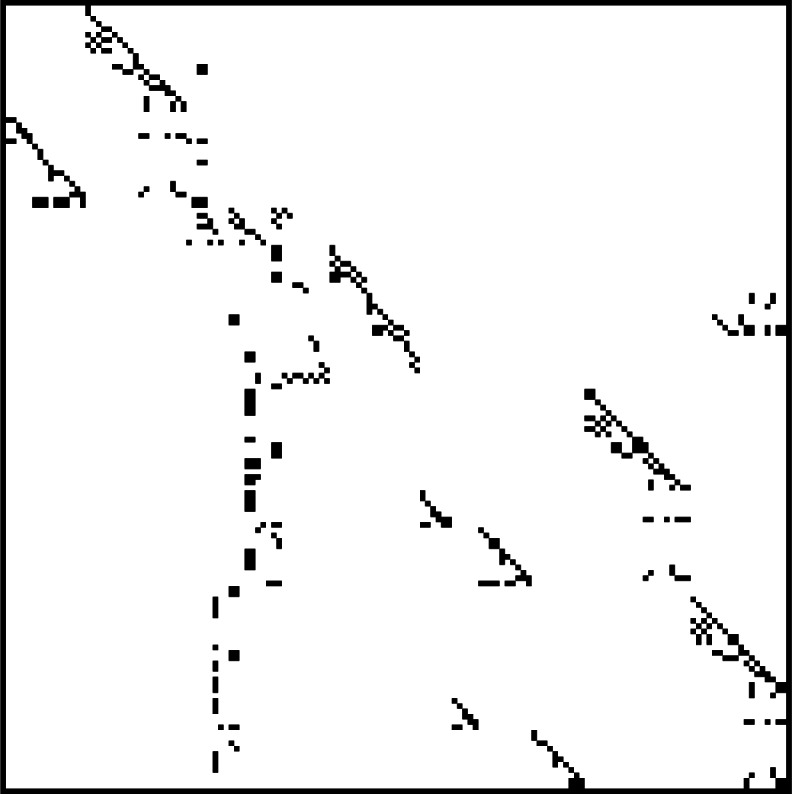
Structure plot of a sparse matrix. Dark spots indicate the position of nonzero matrix elements.

The decomposition, solution and eigenanalysis of systems of linear equations are fundamental problems in scientific computation for which new algorithms and software packages are continually being developed. The study of measures of inherent difficulty for the solution of such problems, so-called *condition numbers*, occupied mathematicians at the NBS INA in the 1950s [10]. Today, linear systems of equations that are represented by sparse matrices remain of paramount importance. A sparse matrix is a matrix in which most elements are zero. Figure 7 is a sparsity plot for such a matrix; the dots show where nonzeros are located. Problems of this type arise in modeling based on partial differential equations, such as in fluid flow and structural analysis. The behavior of algorithms and software for such problems is highly dependent on the sparsity structure and the numerical properties derived from the underlying problem. As a result, in order to make reliable, reproducible and quantitative assessments of the value of new algorithmic developments it is useful to have a common collection of representative problems through which methods can be compared. Researchers in this area have exchanged problem sets of this type informally for some time. One of the difficulties with such collections is that their size and diversity makes them unwieldy to manage and use effectively. As a result, such collections have not been used as much as they should, and matrices useful for testing are not easy to find.

Developments in network communications infrastructure, such as the World Wide Web, have provided new possibilities for improving access to and usability of test corpora of this type. The NIST Matrix Market is such a Web-based repository of matrices for use in the comparative analysis of algorithms and software for numerical linear algebra. More than 500 matrices of size up to 90 449 × 90 449 from a wide variety of applications are made available in the Matrix Market. Matrices are gathered together into sets. Matrices in a set are related by application area or contributed from a single source. Sets are grouped further into collections, such as the well-known Harwell-Boeing collection. Individual matrices may be stored explicitly as dense or sparse matrices, or may be available implicitly via a code that generates them. Matrix generators are either run at NIST remotely via Web-based form, or run locally as a Java applet in a Web browser. In other cases, Fortran code may be downloaded for inclusion in a local testing application. Available matrices are of a wide variety of types, e.g., real, complex, symmetric, nonsymmetric, Hermitian. Some are only representations of nonzero patterns. Others include supplementary data such as right-hand sides, solution vectors, or initial vectors for iterative solvers. We store matrices and associated material one-per-file, in both the Harwell-Boeing format, as well as in a new Matrix Market format. Software for reading and writing such matrices in Fortran, C, and Matlab^2^ are provided.

For each matrix we provide a summary Web page outlining the properties of the matrix and displaying a graphical representation of its properties. Graphics include sparsity plots such as shown above, and three-dimensional representations in Virtual Reality Modeling Language (VRML) format which can be manipulated graphically in a Web browser. Spectral portraits, which illustrate the sensitivity of matrix eigenvalues, are also available in many cases. Similarly, we have developed a Web page for each set that gives its background (e.g., source and application area), references, and a thumbnail sketch of each matrix’s nonzero pattern. We maintain a separate database that contains all of the information on these pages in a highly structured form. This allows us to manipulate the data in various ways; for example, all of the Web pages for matrices and sets are automatically generated from this database. The database also supports both structured and free-text retrieval. The Matrix Market search tool, for example, allows users to locate matrices with particular special properties, e.g., all real symmetric positive definite matrices with more than 10 000 rows and less than 0.1 % density.

The Matrix Market has supported linear algebra researchers and software developers since 1997. About 500 matrices are downloaded from the site each month. Further details can be found in Ref. [7] or at the Web site http://math.nist.gov/MatrixMarket/.

### 4.2 Micromagnetic Modeling

The purpose of micromagnetic calculations is to compute the behavior of the magnetization in a magnetic material in response to a sequence of applied magnetic fields. This kind of modeling is important for the design of magnetic devices such as magnetic recording heads and media, and for the microstructural design of magnetic materials. If micromagnetic calculations are to substitute for physical experiments, the software employed must first be validated. Careful comparison to experimental results is one way to do this. Such a project is quite ambitious, requiring (1) valid solutions for the model equations, (2) good values of materials parameters, and (3) careful experimental design and assessment of experimental errors.

The work we have done is focused solely on the first of these issues. The mathematical model for micromagnetism is derived from the atomic scale physics of electron spin and orbital interactions, and is thought to be valid over length scales large enough that the magnetization can be approximated by a continuous field of three-dimensional vectors of constant magnitude. This micro-magnetic model consists of a set of nonlinear partial differential equations often referred to as *Brown’s equations*.

Taking the model as a given, we set out to test the output of various computational methods. We have done this through development of standard problems to be solved by the micromagnetics community as a whole, and by developing a public micromagnetic code that can serve as a reference and testbed for computational techniques.

Testing the validity of computed results really tests a number of separate but related things:

- the validity of algorithms,
- correct implementation of algorithms (bug free code), and
- valid use of the algorithms.

These three items require a skilled mathematician, a skilled programmer, and a skilled operator familiar with the limitations of the algorithms. Sometimes, but not always, one person is responsible for all three of these skills.

Our experience with standard problems and reference code has been a consequence of our formation and facilitation of the Micromagnetic Modeling Activity Group (*μ*MAG), which was created to work on standard problems in micromagnetics and on publicly available micromagnetic code.

Our contact with the community of researchers in the field of magnetism, and in micromagnetics in particular, has been mainly through the use of “piggy-back” workshops held as evening sessions at major international magnetism conferences. We began by convening a steering committee of representatives from industry, academia and government labs to plan our first workshop. Following this meeting the importance of the steering committee has diminished, and we have been formulating plans based mostly on feedback, often by quick show-of-hands opinion polls at the workshops.

#### 4.2.1 Standard Problems

It is important to achieve a balance in problem definition between over-specification of the problem and lack of focus. In the extreme limit of over-specification, all aspects of solving the problem are determined, and participants have no freedom to select differing solution methods. Effectively, all participants are forced to run the same program, and comparisons of the contributed solutions can reveal only potential compiler or CPU errors. On the other extreme, characterized by lack of focus, participants solve significantly different problems, and again nothing is learned about the validity of the solutions.

Because we want to test solution methods, in our standard problems we have specified the material geometry, material parameters and applied field directions. All other parameters, including the discretization scheme, discretization size, dynamic behavior and the specific applied field values are left open.

For problems where the results are to be published in archival journals, we try to keep the scope of the problem small so that the standard problem results can be a small part of a larger paper containing variations on the problem. Otherwise, we felt that reviewers and editors might fail to see the value of results that had been previously calculated.

Our first standard problem was proposed, specified, and posted before anyone had attempted to solve it. The problem was conceptually simple, and the parameters corresponded to the parameters of working devices. In retrospect, the first standard problem was poorly selected because it proved to be too computationally demanding for participants to compute a valid solution.

Learning from this mistake, we made the second standard problem specification scalable by selection of a parameter value. For small parameter values, the problem was less computationally demanding than for large parameter values. We expected solutions which closely agree for small parameter values and that diverged as the parameter value increases.

The most important consideration in collecting solutions to a standard problem is that the demands on participants time must be kept to a minimum. We have used two methods of collecting solutions. With our first standard problem, we recognized that initial results were disagreeing rather severely. Recognizing that such results could prove embarrassing to individuals, or even to corporations, the results were posted in anonymous fashion on a collection of web pages, so results could be compared without learning the source of any particular solution. Even under the protection of anonymity, lobbying was often required to obtain solutions.

Following our experience with the first standard problem and having proposed two different, simpler standard problems, we switched to publication of standard problem results in regular archival journals. Workshop attendees felt that this would allow researchers to get credit for their work through normal channels. In addition to publication, we requested data from those publishing papers on the standard problems to post on the *μ*MAG web page where results could be compared side-by-side.

#### 4.2.2 Reference Software

The public code project complements the standard problem suite by providing freely available software with source code that can be used to provide expanded detail on solutions to the standard problems, and provide reference results to other problems. It runs on a wide range of machines, and presents a graphical user interface that allows it to be used by the non-specialist. In particular, it is a useful aid to understanding experimental results.

In developing reference micromagnetic software we had several goals in mind. The code needed to be powerful and flexible enough for in-house research, and to provide sample results for the standard problems. On the other hand, an issued raised in the first MAG meeting was the importance of having a code available that experimentalists could use to help interpret their results, without a major investment of time to learn to use the code. We also wanted a modular code that could be used as a development platform for new researchers in the field of micromagnetics.

To provide portability and an easy to use graphical interface, we decided to write the user interface code in the Tcl/Tk scripting language, while the core of the micromagnetic solver would be written in C++ for modularity and extensibility. To make the code widely available, we created a web site where regular alpha and beta releases of both full source code and executables would be placed.

#### 4.2.3 Results

The activities of MAG, including complete standard problem specifications and results are documented at http://www.ctcms.nist.gov/~rdm/mumag.html. The public reference code is available for download at http://math.nist.gov/oommf/.

**Fig. 8:**
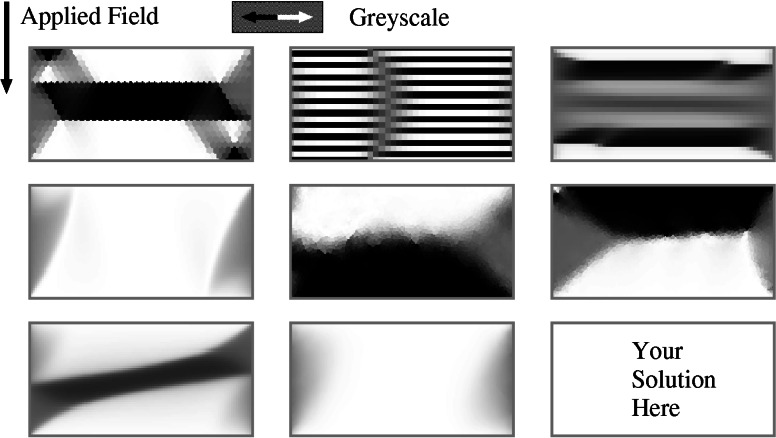
Anonymously submitted solutions to Standard Problem #1. Each image depicts the horizontal component of the magnetization in the zero field state obtained after application of a large field 1° from the vertical.

Our first attempt at a standard problem encountered a number of difficulties. The problem involves calculating the domain patterns and hysteresis loops for a 1 *μ*m × 2*μ*m rectangle of material, 20 nm thick with materials parameters set to mimic Permalloy (Ni_80_Fe_20_). At first this appeared to be a reasonable problem. Problems having a similar geometry were being solved in industry in modeling of recording heads, but the hysteresis loop calculations requested for the standard problem proved to be much more demanding than the calculations of reversible behavior used for the recording heads. A subset of the collected results are shown in Fig. 8. An additional result is published in Ref. [30]. The disparity in the computed results is believed to be due to a number of factors:

- Proximity of specified field values to critical values. The maximum field requested was very close to the field required for complete saturation, and applying the field 1° off axis was insufficient to break the symmetry of the problem
- Large problem dimensions relative to intrinsic length scales. The exchange length for Permalloy is approximately 5 nm. The number of computational grid cells required to discretize down to this length scale was prohibitive.

**Fig. 9:**
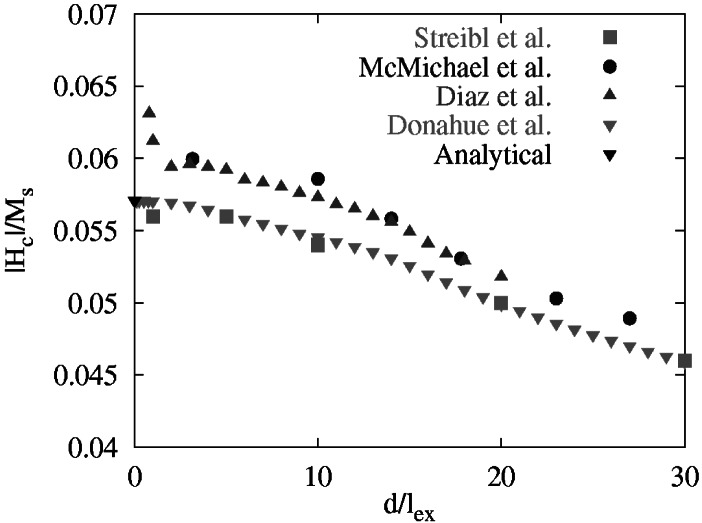
Coercive field as a function of the bar width for Standard Problem #2. The labels refer to the authors of Refs. [3, 12, 22, 25].

Standard Problem #2 is a bar of magnetic material, with aspect ratios 5:1:0.1. The material is specified by magnetization *M*_s_ and exchange stiffness parameter *A*. The intrinsic length scale, the exchange length, is given by $\delta =\sqrt{2A/\mu_{0}M_{s}^{2}}$. Magnetocrystalline anisotropy is set to zero. All length scales can be expressed in unit of δ and all fields can be expressed in units of *M*_s_. With the applied field oriented in the [1,1,1] direction relative to the principle axes of the bar, the problem is to calculate the remnant magnetization and coercivity of the bar as a function of the bar width. The problem is expected to become progressively more challenging as the bar dimensions are increased relative to *δ*. Results have been published in the open literature [3, 12, 22, 25] including an analytical solution for the limit of small dimensions [12]. A representative plot is shown in Fig. 9.

**Fig. 10:**
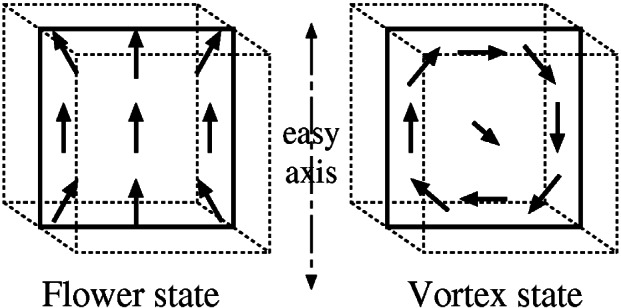
Schematic drawings of the expected “flower” and “vortex” magnetization states for Standard Problem #3.

Standard Problem #3, like problem #2, involves an object with variable dimensions, but is more suitable for a 3D code. The object is a cube with magnetization, *M*_s_, exchange stiffness parameter, *A*, and magnetocrystalline anisotropy constant $K_{u}=0.1\times \frac{1}{2}\mu_{0}M_{s}^{2}$, with the easy direction parallel to a principle axis of the cube. There is no applied field. For very small cubes, the minimum energy state is expected to be nearly uniform magnetization, and for a large cube the minimum energy state is expected to involve a number of domains. The problem is to find the cube edge length, *L*, such that the nearly uniform “flower” state has the same energy as a “vortex state” (Fig. 10). Solutions for this problem agree well, ranging from *L* = 8.469*δ* to *L* = 8.52*δ*, with one paper published in the open literature [29] and the others described on the web site listed above.

The problem specification assumes that the “flower” and “vortex” states are the two lowest energy states for the cube. However, in one of the submitted solutions, the existence of another “twisted flower” state is described that has the lowest energy near *L* = 8.5.

Standard Problem #4 is a “proposed” problem, currently posted for comments from the community. It is primarily intended as a test for dynamic micromagnetic calculations. The material is a 500 nm × 125 nm rectangle of material, 3 nm thick, with parameters designed to mimic Permalloy. The dynamics of the magnetization have been specified as the Landau-Lifshitz-Gilbert equation.

Starting with magnetization in a specified zero-field “s-state,” fields are applied instantaneously to reverse the magnetization, and the evolution of the magnetization is traced as the magnetization comes to equilibrium in its new state. Calculations for two switching fields are specified, applied 170° and 190° from the long axis of the rectangle of material. Because the s-state is asymmetric, for one of these fields the magnetization will reverse by rotating in the same direction throughout the sample. The computation is expected to be more difficult for the other applied field, since preliminary computations have shown that the magnetization initially rotates in different directions in different parts of the sample, creating vortices and domain walls that are more difficult to resolve.

#### 4.2.4 Outcomes

We have developed several standard problems in micromagnetics, and have enlisted the help of the micro-magnetics community in generating results to these standard problems. As our experience has increased, we have become better at proposing tractable, well-defined problems. As a result, we have been able to shift from collecting anonymous solutions to the problems, where the identity of the solution author is protected, to solutions that are subject to peer review and published in the normal way. This transition is important since it allows the problem solvers to get credit and financial support for their work through normal channels. The standard problems are now in a state where they can be used in a limited way for their intended purpose, to detect errors in micromagnetic computer software.

The first public release of the reference micromagnetic software occurred in January 1998. The software was developed by Michael Donahue and Donald Porter, with some early contributions by Robert McMichael. We have released upgrades on a regular basis since that time. The documentation, which is included with each release and is available on the web site in online form, has been published as a NIST report [11]. The software runs on a wide variety of Unix and Windows computers, and has contributed to at least 10 papers in refereed journals. We have also used the reference code to provide solutions to the standard problems. This is especially useful as interested parties can determine additional details about the solutions not included in the reports by downloading the software and replicating the results. This is also a good practice exercise for learning to use the code.

Results from the standard problems also feed back and influence the public code. For example, the first three submitted solutions to Standard Problem #2 were Streibl, McMichael, and Diaz (refer to Fig. 9). We expected the solutions to agree for small values of *d*/*l*_ex_, and although close, there appeared to be a systematic disagreement between the Streibl results and the other two. We examined our results (McMichael) more closely, and determined that there was a bias in the calculation of the demagnetization field near the edges of the bar [12]. We implemented an improved demagnetization module, and submitted new results (Donahue) that agree closely with the Streibl results, and the analytic result in the small particle limit.

## 5. Digital Library of Mathematical Functions

In Sec. 2.3 we described the development of the NBS *Handbook of Mathematical Functions*. The functions whose properties were laid out in this reference work continue to play a critical role in applied mathematical modeling. As a result, practitioners still need ready access to a reliable source of information about mathematical functions, which accounts for the Handbook’s continued popularity. Nevertheless, it is now out-of-date in many respects. Since its publication, numerous advances in related fields of mathematics have been made:

- New functions have entered the realm of practical importance, e.g., q-series.
- New fields of application have emerged, e.g., in nonlinear dynamics.
- Analytical developments have occurred, e.g., in asymptotics.
- New properties, e.g., integral representations and addition formulas, have been discovered.
- Numerical developments, e.g., interval analysis and Padé approximations, have occurred.
- Computer algebra and symbolic processing have come into wide use.
- An enormous increase in computing power has made obsolete standard numerical processes of the 1950s, such as table-making and interpolation, while increasing the value of others.
- Comprehensive software packages have been constructed for working and computing with functions.

At the same time, dissemination of information is being revolutionized by the rapid development of the Internet and World Wide Web. A modern successor to the Handbook should provide new capabilities unavailable in print media, such as:

- Generic representation of mathematical entities such as formulas, graphs, tables and diagrams.
- Advanced search, with the ability to locate formulas based on mathematical subexpressions.
- Downloading of mathematical entities into document processors.
- Importing of formulas directly into symbolic computing systems.
- Continuous updating to incorporate corrections, additions and extensions.
- Maintenance of communication channels between users and developers, with a public record of usage.
- Support for external application modules to provide tutorials, application notes or research monographs in fields that use mathematical functions.
- Recommendations of algorithms and software for computing functions, with links to sources.
- Generation of numerical tables and graphs for user-specified ranges of input.

NIST has begun the process of completely rewriting the Handbook for presentation as an on-line resource with many of these features. The result will be the NIST Digital Library of Mathematical Functions (DLMF). The project entails (a) gathering all pertinent mathematical information, (b) constructing a state-of-the-art reference database with all necessary tools for long-term maintenance, (c) presenting validated reference information on the Web, and (d) developing application modules in quantum mechanics, electromagnetism, and an adaptive learning system for mathematical functions.

The DLMF project is being managed by four principal editors at NIST: Daniel Lozier, Frank Olver, Charles Clark, and Ronald Boisvert. They are assisted by a panel of 10 associate editors representing expertise in special functions, numerical analysis, combinatorics, computer algebra, physics, chemistry, and statistics. The DLMF will have 38 chapters:

1. Mathematical and Physical Constants
2. Algebraic and Analytical Methods
3. Asymptotic Approximations
4. Numerical Methods
5. Computer Algebra
6. Elementary Functions
7. Gamma Function
8. Exponential Integral, Logarithmic Integral, Sine and Cosine Integrals
9. Error Functions, Dawson’s Integral, Fresnel Integrals
10. Incomplete Gamma Functions and Generalized Exponential Integral
11. Airy and Related Functions
12. Bessel Functions
13. Struve Functions and Anger-Weber Functions
14. Confluent Hypergeometric Functions
15. Coulomb Wave Functions
16. Parabolic Cylinder Functions
17. Legendre Functions and Spherical Harmonics
18. Hypergeometric Functions
19. Generalized Hypergeometric Functions and Meijer G-Function
20. q-Hypergeometric Functions
21. Classical Orthogonal Polynomials
22. Other Orthogonal Polynomials
23. Elliptic Integrals
24. Theta Functions
25. Jacobian Elliptic Functions
26. Weierstrass Elliptic and Modular Functions
27. Bernoulli and Euler Numbers and Polynomials
28. Zeta and Related Functions
29. Combinatorial Analysis
30. Functions of Number Theory
31. Statistical Methods and Distributions
32. Mathieu Functions and Hill’s Equation
33. Lame Functions; Spheroidal Wave Functions
34. Heun Functions
35. Painleve Transcendents
36. Integrals with Coalescing Saddles
37. Wavelets
38. 3j, 6j, 9j Symbols

The second through fifth chapters will provide background material in mathematical and numerical analysis. The remaining chapters deal with individual functions or classes of functions. An emphasis will be placed on a concise presentation of the mathematical properties of the functions, including formulas, visualizations, methods of computation, and representative applications. Pointers to state-of-the-art software for computing the functions will also be supplied. Detailed tables of function values, which occupied more than half of the original Handbook, will not be included The last six chapters contain material on functions which were not represented in the original Handbook, and all the chapters are enlarged. Tables aside, the DLMF will contain twice the material found in the original Handbook.

The technical material is being developed by some 50 external participants. Of these, authors under contract to NIST will complete a survey of the literature as the basis for their chapters. After chapters are approved by the Editorial Board they will be carefully checked by independent external validators, also under contract to NIST.

**Fig. 11:**
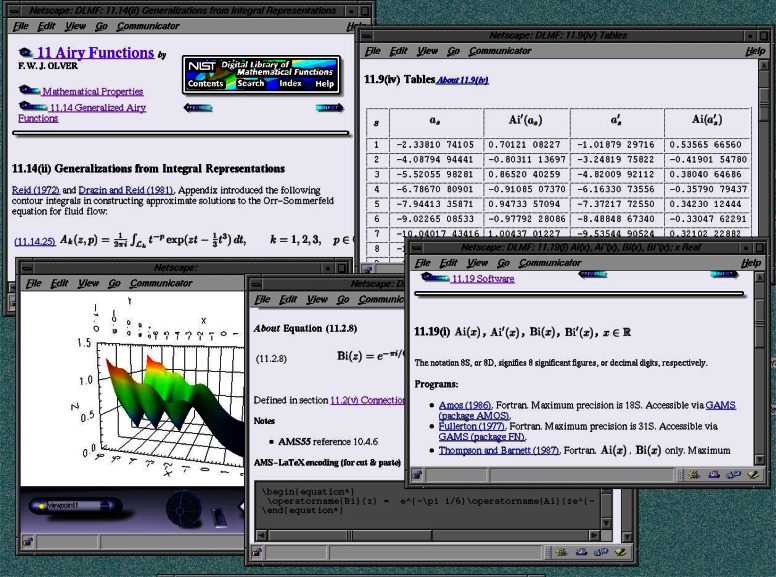
Windows illustrating the capabilities of the Digital Library of Mathematical Functions.

The DLMF will be made available in a highly interactive Web site maintained by NIST (see Fig. 11). Each labeled item (e.g., section, formula, table) will have metadata associated with it, both to aid in searching, and to provide readers with further information such as links to original references and generalizations. Interactive tools for visually exploring functions will be supplied as Java applets or as VRML worlds. Providing certified tables of function values on demand for each of the functions in the DLMF is beyond the scope of the current project. However, this is recognized as a need, and it will be demonstrated for several of the functions in the DLMF.

A prototype of the Digital Library can be inspected at http://dlmf.nist.gov/. Completion of the system is expected in 2002.

## 6. Future Trends in Mathematics at NIST

The widespread availability of substantial computational power will increase the demand for mathematical and computational modeling. As more and more people attempt to exploit such methodology, there will be greater need for specialized, but flexible, computational problem-solving environments for science and engineering applications [31]. These will be built from mathematical components which must have a higher degree of reliability than those in common use today. Mathematical research will be needed in the development of fast, reliable, adaptive and self-validating algorithms for a wide variety of problems. As the development of mathematical software components moves from the research community to the commercial sector there will also be a critical need for techniques and tools to assess the accuracy and reliability of mathematical systems and components. NIST is a natural home for the development of measurement technology in this area.

Increased computational modeling capabilities will have an even greater impact on future NIST measurement programs. As higher fidelity models are proposed and efficient solution techniques developed, there comes the real possibility of replacing many physical measurements by *virtual measurements* performed on mathematical models. Before this can occur, however, substantial efforts must be made to more carefully characterize models and sources of error so that the precision and accuracy of virtual measurements can be quantitatively assessed in the same way as physical measurements. Of course, such technology would not make experiment-based metrology obsolete. Instead, experimental measurements would be targeted to the calibration and validation of mathematical models.

## Footnotes

1 A publication of the IEEE Computer Society and the American Institute of Physics.

2 Certain commercial equipment, instruments, or materials are identified in this paper to foster understanding. Such identification does not imply recommendation or endorsement by the National Institute of Standards and Technology, nor does it imply that the materials or equipment identified are necessarily the best available for the purpose.
